# Acute high-fat high-sugar diet rapidly increases blood-brain barrier permeability in mice

**DOI:** 10.1016/j.jnha.2025.100574

**Published:** 2025-05-16

**Authors:** Este Leidmaa, Andreas Zimmer, Valentin Stein, Anne-Kathrin Gellner

**Affiliations:** a Institute of Molecular Psychiatry, Medical Faculty, University of Bonn, Venusberg-Campus 1, 53127 Bonn, Germany; b Department of Physiology, Medical Faculty, University of Tartu, 19 Ravila Street, Tartu 50411, Estonia; c Institute of Physiology II, Medical Faculty, University of Bonn, Nussallee 11, 53115 Bonn, Germany; d Department of Psychiatry and Psychotherapy, University Hospital Bonn, Venusberg-Campus 1, 53127 Bonn, Germany

**Keywords:** Western diet, Palatable food, Vascular leakage, BBB integrity, Drug delivery

## Abstract

The blood-brain barrier (BBB) maintains brain homeostasis by protecting the brain from pathological stimuli and controlling the entry of physiological substances from the periphery. Consequently, alterations in BBB permeability may pose a threat to brain health. Long-term consumption of a high-fat high-sugar/Western diet (HFD) is known to induce BBB dysfunction. However, nothing is known about the immediate effects of acute HFD consumption on the BBB. Using spectrophotometry and *in vivo* 2-photon microscopy in mice, we demonstrate region-specific BBB leakage already after 1 h of HFD for low- and high-molecular-weight tracers. Acute HFD also significantly increased BBB permeability to the anticancer drug doxorubicin. These previously unknown effects of acute HFD in mice may have far-reaching implications for the clinical use of drugs depending on the dietary habits of the patient, and might inform future studies on drug transport to the brain.

## Graphical abstract

**Figure:**
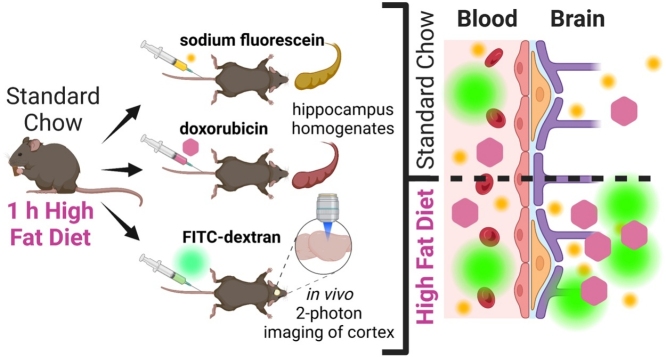

## 1 Introduction

The blood-brain barrier (BBB) is a boundary between the bloodstream and the brain that tightly filters the passage of substances and nutrients from the blood to the brain. The BBB is clinically relevant in protecting the brain from toxins and pathogens, while maintaining a constant balance of water, nutrients, and hormone levels in the brain. The effective BBB also has a downside; many potential new drug candidates for mental and neurological diseases do not readily cross the BBB [1].

Alterations in BBB permeability can be induced by several physiological or pathological systemic and central states [2]. In humans and mice alike, pathological triggers can be either short and strong, for example stroke, bacterial infection, trauma, or milder and longer-lasting like autoimmune disease, neurodegeneration, chronic stress, and obesity [[3], [4], [5], [6], [7], [8], [9]]. Chronic consumption of a high-fat high-sugar/Western diet (HFD) can lead to obesity and related sequelae. Importantly, HFD has also been shown to induce BBB dysfunction [3,10]. We were the first to show the effects of a very short HFD treatment of 1 h and demonstrated that this acute HFD interfered with central mechanisms of feeding control [11]. The impact of acute HFD on BBB permeability is completely unknown. Based on our previous observations, we now wondered whether such a short-term treatment would also affect BBB permeability. Surprisingly, using different methods, including *in vivo* microscopy, we demonstrate that the permeability of low- and high-molecular weight tracers, or the chemotherapeutic drug doxorubicin, is dramatically increased by this treatment.

## 2 Materials and methods

### 2.1 Mice and metabolic treatments

Adult male mice (C57BL/6N) aged between 4 and 10 months, with each cohort having a similar within-cohort age range, were used for all experiments (Supplementary Table S1). Animals were single-housed throughout the *in vivo* imaging experiments starting after surgery, or for a minimum of 2 weeks prior to all other experiments. Mice were fed *ad libitum* and were housed under a 12:12 -h light/dark cycle (light phase from 7 am to 7 pm) at a constant temperature (22 °C). Experiments were carried out in the beginning light phase. All experiments followed the guidelines of the German Animal Protection Law and have been approved by the government of North Rhine Westphalia (Local Committee for Animal Health, LANUV NRW).

Mice were weighed at the start and the end of each treatment period. Mice were offered a free choice high-fat/high-sugar diet (HFD, EF 12451, ssniff Spezialdiäten, Germany) and standard chow (V-1534300, ssniff Spezialdiäten), ∼10 g each, or only standard chow for the control group for either 1 h or 24 h. HFD contained 4.615 kcal/g and had 45% metabolizable energy from fat, 35% from carbohydrates (21% from sugar), and 20% from protein. Standard chow had 3.89 kcal/g and contained 9% of energy from fat, 58% from carbohydrates (4% of sugar), and 33% from protein. The amount of food consumed was measured. No pre-treatment fasting period was used. Of note, all mice chose to eat HFD and not standard chow during our treatment periods. To avoid neophobia, all mice were habituated to HFD by receiving a single pellet one day before its first introduction in the experiment, which ensured that the mice would promptly consume HFD during the 1-h exposure (average intake 0.8 g ± 0.4 g SD, 2.9 % ± 1.4 % SD of their body weight) [11]. Water was available *ad libitum* throughout the treatment periods.

### 2.2 Sodium fluorescein assay

The protocol was modified from Kaya & Ahishali [12]: Mice were anesthetized (medetomidine 0.5 mg/kg, midazolam 5 mg/kg body weight i.p.) after 1 h of HFD or control treatment. Sodium fluorescein (NaF, F6377, Sigma) was dissolved in saline (30 mg/ml) and slowly injected into the tail vein according to body weight (120 mg/kg). After a circulation time of 30 min, mice were given a euthanizing dose of ketamine/xylazine (240 mg/kg, 32 mg/kg body weight i.p.). The mice were transcardially perfused with 50 ml cold phosphate-buffered saline (PBS, pH 7.4) at 10 ml/min. The brain was removed, and the cortex, hippocampi, cerebellum, and hypothalamus were dissected, weighed, and snap-frozen in liquid nitrogen. All further steps were carried out ensuring light protection of the samples. Tissue was thawed on ice, homogenized in 150 µl of PBS after which 150 µl of 60% trichloroacetic acid (T0699, Sigma) was added, and the homogenates were vortexed for 2 min before incubation for 30 min at 4 °C. Samples were centrifuged at 18,000 g at 4 °C for 10 min and 150 µl of supernatant per sample were measured for absorbance at 440 nm with a plate reader (Infinite 200 Pro, Tecan) in a 96-well microplate. Tissue content of NaF was quantified from a linear standard curve derived from the dye and expressed in nanogram per milligram of brain tissue.

### 2.3 Cranial window surgery and 2-photon *in vivo* imaging

Mice were deeply anesthetized (medetomidine 0.5 mg/kg, midazolam 5 mg/kg, fentanyl 0.05 mg/kg body weight i.p.) and received carprofen 5 mg/kg s.c. for perioperative analgesia. A craniotomy 3−4 mm in diameter was carefully drilled over the left somatosensory cortex. A round glass coverslip 5 mm in diameter was placed and sealed with cyanoacrylate and dental acrylic over the craniotomy. A custom-made plastic bar was attached to the parietal bone contralateral to the trepanation for fixation during microscopy. Anesthesia was antagonized (atipamezole 2.5 mg/kg, flumazenil 0.5 mg/kg, naloxone 1.2 mg/kg body weight i.p.) and mice allowed to return to full alertness and normal mobility under a warming lamp. Carprofen (5 mg/kg body weight) was applied for analgesia 1x/day subcutaneously for 3 days starting 12 h post-surgery.

After a minimum recovery period of 25 days mice were slightly anesthetized (medetomidine 0.5 mg/kg, midazolam 5 mg/kg body weight i.p., toe pinch slightly positive) to ensure immobilization during the imaging session. 70 kDa FITC-dextran (Sigma 4695/100 mg/F) was dissolved in saline (50 mg/ml) and 100 µl were slowly injected into the tail vein. A custom-built 2-photon microscope equipped with a Chameleon Vision S laser (Coherent) and a water immersion objective lens (40×, NA 0.8, Olympus) was used for *in vivo* imaging. Images were acquired using ScanImage software (MBF Bioscience). Excitation wavelength was tuned to 910 nm for imaging of FITC-Dextran. Time-lapse stacks (xyz-dimension 334 × 334 × 40 µm, xy-resolution 0.33 µm/px, step size 2 µm) of a region of interest (ROI) containing a vascular network were recorded every 3 min for 30 min at a frame rate of 0.43 Hz. After imaging, anesthesia was antagonized with atipamezol 2.5 mg/kg and flumazenil 0.5 mg/kg body weight i.p and mice were allowed to fully recover in their home cage under a warming lamp. Imaging sessions of the same animal were conducted with a minimum interval of 5 and a maximum interval of 12 days. Treatments before the imaging sessions were done in a pseudorandomized order and the area with the region of interest was re-identified each time using a 4× objective. By this, every animal served as its own control except for one which accidentally lost its cranial window before the standard chow condition. The results of one mouse after 1 h of HFD had to be excluded as a technical outlier. Number of imaging sessions per animal ranged between 2 and 6 (75% of mice being imaged 3−4 times), with repetitions becoming necessary due to randomly occurring technical problems during imaging.

### 2.4 Image analysis

**Fig. 1:**
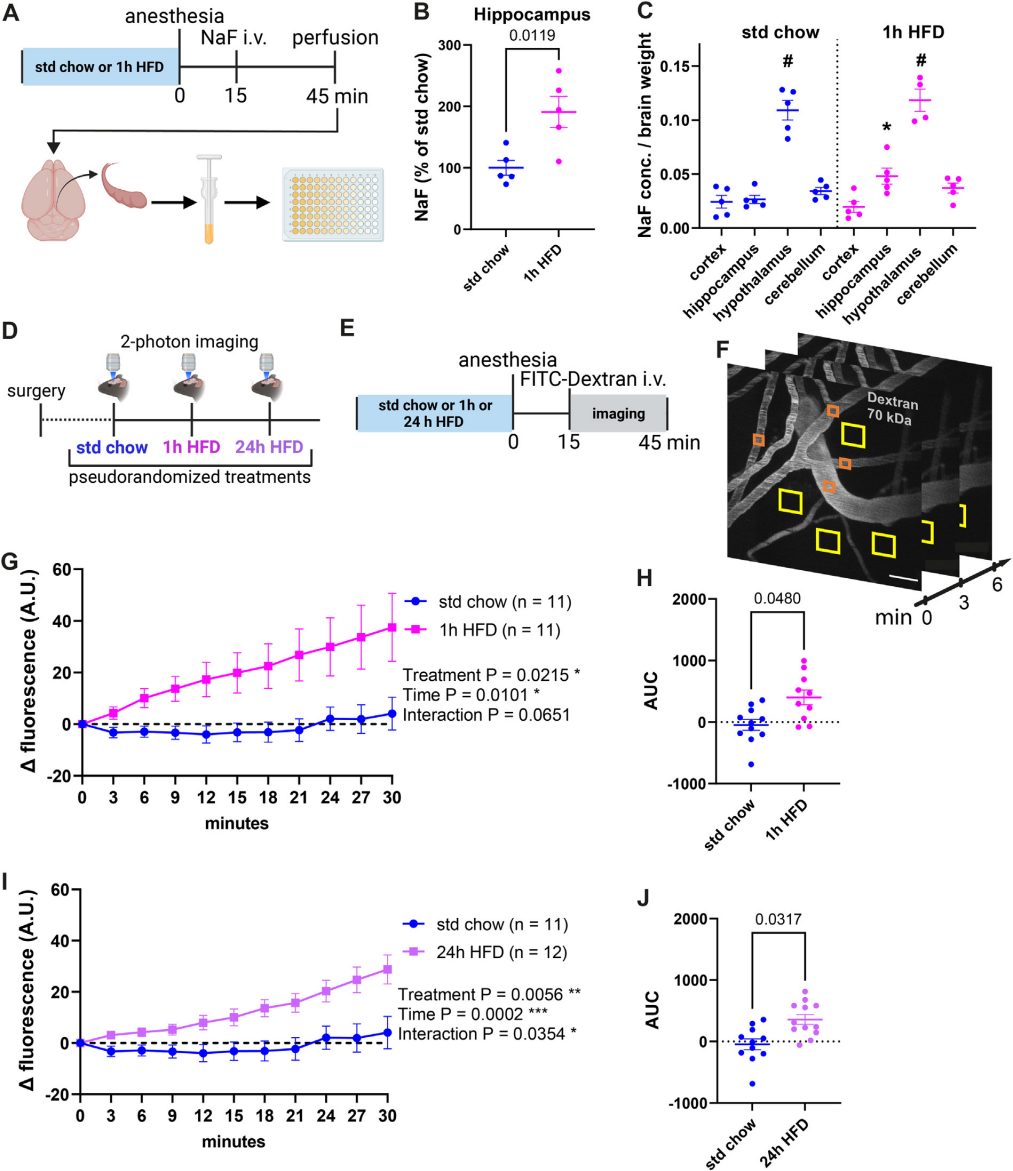
Leakage of the small-molecular-weight tracer sodium fluorescein (NaF, 376 Da) and a high-molecular-weight tracer FITC-dextran (70 kDa) from blood into the brain tissue was increased already after 1 h of high-fat high-sugar diet (HFD) exposure. **(A**) Scheme showing the design of the experiment with NaF and workflow of the tissue processing with dissection of the regions of interest, homogenization, and fluorometric measurements. **(B)** Relative NaF concentrations in hippocampal homogenates were elevated (t(8) = 2.58, *p* = 0.0327) after 1 h of HFD compared to control mice on standard chow. **(C)** The hypothalamus showed the highest absolute NaF leakage into the brain tissue compared to the other regions both in control condition (F(1.561, 8.325) = 46.08, *p* < 0.0001) and after 1 h HFD (F(1.428, 5.236) = 47.53, *p* = 0.0006). *P* < 0.05, * hippocampus compared to cortex, # hypothalamus compared to all other brain areas. Error bars represent mean ± SEM. **(D)** Scheme showing the design of the experiment with repeated *in vivo* imaging of dextran-filled cortical vasculature and perivascular space in the somatosensory cortex (S1) after 3 pseudorandomized treatments of either standard chow, 1 h, or 24 h HFD**. (E)** Workflow of treatment and dextran injection before an imaging session. **(F)** Example of dextran-filled vasculature and analysis of intra- (orange squares) and extravascular (yellow squares) fluorescence in maximum projections of time-lapse z-stacks, scale bar 50 µm. **(G)** Acute 1 h HFD significantly increases extravascular FITC-dextran levels in the somatosensory cortex over the time course of imaging (treatment F(1, 11) = 7.173, *p* = 0.0215, time F(1.525, 16.78) = 6.897, *p* = 0.0101, time x treatment interaction F(1.241, 10.92) = 3.989, *p* = 0.0651) and the leakage was also significantly higher (t(8) = 2.33, *p* = 0.0480) when expressed as area under the curve (AUC) **(H)**. **(I)** Exposure to HFD for 24 h increased the leakage of FITC-dextran into the somatosensory cortex significantly for analysis over time (treatment F(1, 11) = 11.81, *p* = 0.0056, time F(2.407, 26.47) = 10.97, *p* = 0.0002, time × treatment interaction F(1.53, 15.14) = 4.593, *p* = 0.0354) and when expressed as AUC (t(10) = 2.49, *p* = 0.0317) **(J).** Error bars represent mean ± SEM.

Preprocessing and analysis of the *in vivo* 2-photon images were done with Fiji/ImageJ2 (2.9.0). The z-stacks from all the time-points were transformed into maximal projections which were then added together into a time-stack (10 frames in 3-minute steps). Image registration was done with “Linear Stack Alignment with SIFT” and expected transformation “Translation”. Four rectangular regions of interest (ROIs) of 16.5 × 16.5 µm were placed to the intravascular space with at least a 2 µm distance from vessels, and 4 ROIs of 33 × 33 µm were placed on the vessels as a control measure (Fig. 1F). Fluorescence intensity (mean gray value) was measured for all ROIs. Data from the 4 extravascular and 4 intravascular ROIs were averaged respectively per imaging session into one set of time-lapse values. All the subsequent time points were normalized to the first time point (T_n_-T_0_), which served as the baseline. Areas under the curve (AUC) were additionally calculated for each imaging session from every animal using GraphPad Prism (Version 9.4.1).

### 2.5 Doxorubicin assay

The protocol was modified from Bachur and colleagues [13]. Mice were anesthetized (medetomidine 0.5 mg/kg, midazolam 5 mg/kg body weight i.p.) after 1 h of HFD or control treatment. Doxorubicin-hydrochloride (44583, Sigma) was dissolved in saline (1.25 mg/ml) and slowly injected into the tail vein according to body weight (5 mg/kg). After a circulation time of 60 min, at which peak concentrations of doxorubicin can be expected [14], mice were given a euthanizing dose of ketamine/xylazine (240 mg/kg, 32 mg/kg body weight i.p.). The mice were transcardially perfused with 50 ml cold phosphate-buffered saline (PBS, pH 7.4) at 10 ml/min. The brain was removed, and cortex, hippocampi, cerebellum, and hypothalamus were dissected, weighed, and directly homogenized in 10 volumes of 0.3 N HCl in 50 % ethanol. All further steps were carried out ensuring light protection of the samples. Homogenates were incubated at 4 °C for 24 h before centrifugation at 16,000*g* for 25 min at 4 °C. Supernatants were collected and 100 µl of each sample measured with a spectrophotometer (Nanodrop 2000c, Thermo Scientific). Doxorubicin concentrations were determined from absorbance at 480 nm and a linear standard curve derived from the compound, and expressed in nanogram per gram of brain tissue.

### 2.6 Statistics

Data was analyzed and plotted with GraphPad Prism, Versions 9.4.1. and 9.5.0. Drawings were created with BioRender.com. The numbers of animals/samples are indicated in the legends. Statistical outliers were identified using the ROUT method (Q = 1 %). The Shapiro-Wilk test was used to estimate whether the data was normally distributed. Statistical significance was assessed using Student's *t*-test for parametric data or Mann–Whitney test for nonparametric data. One-way ANOVA was used for matched values/Mixed model analysis with Geisser-Greenhouse correction and Tukey’s multiple comparison test was used to analyze more than two groups. Repeated measures 2-way ANOVA/Mixed model analysis with Geisser-Greenhouse correction with Sidak’s multiple comparison tests were used to evaluate time-lapse values.

## 3 Results

We injected the low molecular weight tracer sodium fluorescein (NaF, 376 Da) intravenously in anesthetized mice after they had eaten HFD for 1 h and subsequently analyzed the levels of NaF in brain homogenates (Fig. 1A). As shown in Fig. 1B, HFD exposure resulted in a significant increase in tracer fluorescence specifically in the hippocampus, thus indicating that the BBB permeability was increased after acute HFD. No acute effect of HFD was detectable in samples from cortex, cerebellum, and hypothalamus by this method (Suppl Fig. S1B–D). Of note, the hypothalamus with its specialized fenestrated BBB [15] showed markedly higher fluorescence levels compared to the other brain regions in both treatment groups (Fig. 1C).

We next investigated BBB leakage of the high molecular weight tracer FITC-dextran (70 kDa) using 2-photon *in vivo* imaging through a cranial window over the somatosensory cortex after the tracer’s intravenous injection in anesthetized mice (Fig. 1D–F). The somatosensory cortex is known to be activated by the perception and processing of stimuli such as palatable food in humans [16,17]. This *in vivo* imaging method provides a high spatial resolution, allows for repeated measurements over several time points in each individual, and enables within-subject comparison. First, we measured the effect of 1 h of HFD consumption *versus* standard chow within 30 min after tracer injection. Whereas mice fed with a standard chow showed no increase of extravascular FITC-dextran during the 30-minute observation period, we detected a significant leakage of FITC-dextran into the extravascular space in mice exposed to HFD. The control measure of intravascular fluorescence decreased over time as expected (Suppl Fig. S1E). We repeated the analysis after an exposure to a 24-h HFD treatment, which is known to increase BBB leakage in the hippocampus [18]. Strikingly, the 24-h treatment increased the BBB leakage to a similar extent as the 1-h treatment when compared over the time course (Fig. 1G, I) and when the cumulative areas under the curve (AUC) were analyzed (Fig. 1H, J). Direct comparison also showed no significant difference between the two treatment groups (Suppl Fig. S1F). Together, these findings indicate that the 1-h HFD already produced a comparable effect to that of a prolonged treatment.

**Fig. 2:**
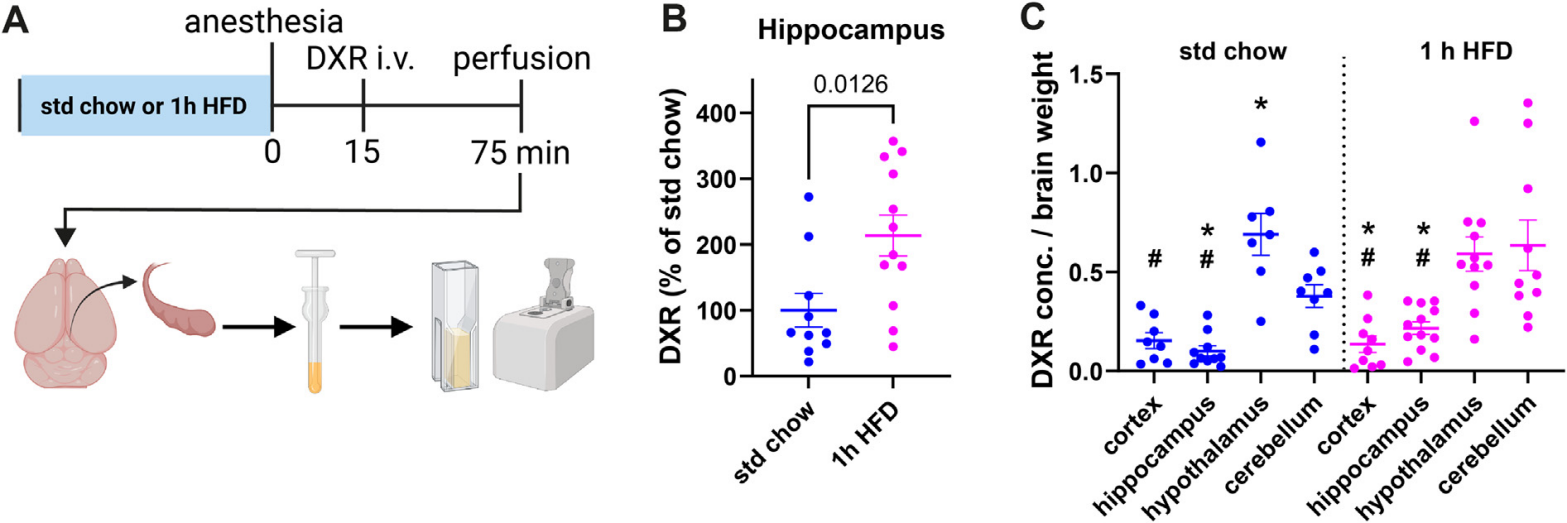
Leakage of the chemotherapeutic agent doxorubicin (DXR, 580 Da) from blood into the brain tissue was increased in the hippocampus after 1 h of high-fat high-sugar diet (HFD) exposure. **(A)** Scheme showing the design of the experiment and workflow of the tissue processing with dissection of the regions of interest, homogenization, and spectrophotometric measurements. **(B)** DXR was elevated in the hippocampus (t(20) = 2.74, *p* = 0.0126) after 1 h of HFD normalized to control mice on standard chow. **(C)** Hypothalamus and cerebellum showed the highest absolute NaF leakage into the brain tissue compared to the other regions both in control condition (F (3, 29) = 20.21, *p* < 0.0001) and after 1 h HFD (F (3, 38) = 9.911, *p* < 0.0001) **(F)**. *P* < 0.05, * hippocampus compared to cerebellum, # hypothalamus compared to all other brain areas. Error bars represent mean ± SEM.

We next sought to determine if the unexpectedly rapid modulation of the BBB function could be clinically relevant. For this purpose, we tested the BBB penetrance of doxorubicin-hydrochloride, a widely used chemotherapeutic agent which is effective against various cancers [19]. Doxorubicin-hydrochloride has a relatively poor BBB penetrance, which is an advantage in the treatment of non-brain tumors. For brain tumors, however, an improved BBB permeability would be advantageous, and this has been the aim of different studies [14,20]. The intrinsic fluorescence of doxorubicin can be used as a proxy for its entry to the tissue. When we injected doxorubicin-hydrochloride (580 Da) intravenously after 1 h of HFD exposure (Fig. 2A), fluorescence was significantly increased in hippocampal homogenates 1 h after treatment compared to the standard chow condition. (Fig. 2A and B). As we had previously observed for NaF (376 Da), no differences were detected in the cortex, cerebellum, and hypothalamus (Suppl Fig. S1B–D). Again, the hypothalamus showed higher fluorescence levels compared to the other brain regions in both treatment groups, while the increase seen in the cerebellum was unique to doxorubicin (Fig. 2C).

## 4 Discussion

In this paper, we demonstrate an increased BBB permeability after acute exposure to HFD, by analyzing the uptake of low-molecular-weight tracers in hippocampal homogenates, as well as the leakage of a high-molecular-weight tracer into the cortex using 2-photon *in vivo* microscopy. The magnitude of BBB permeability was similar after a 1-h treatment and a 24-h exposure, indicating that an acute treatment already produced a maximal effect. Our findings might have clinical relevance, as a 1-h treatment also increased BBB permeability for the chemotherapeutic drug doxorubicin. This finding indicates that certain pharmacotherapies may be acutely affected by dietary factors in unexpected ways.

Many studies have addressed changes in BBB permeability under pathological conditions but also upon physiological stimuli, including high-caloric sugar and fat-containing diets [2,21]. This is particularly important when considering the global spread of such an unhealthy diet. Indeed, several studies demonstrated that longer periods of HFD exposure, 24 h and more, increased tracer penetration into brain tissue in rodents [3,10,18]. Unfortunately, the molecular and cellular mechanisms underlying the effects of HFD on the BBB remain elusive.

The fact that we detected rapid leakage in hippocampal homogenates but not in other brain regions led to using a method with much higher spatial resolution. Two-photon *in vivo* microscopy allows repeated analysis of the cortical BBB in different conditions, as we have previously demonstrated [22]. Indeed, with this high-resolution method, we detected rapid and robustly increased extravasation of FITC-dextran, a high-molecular-weight tracer as large as plasma albumin (70 kDa), into the somatosensory cortex after 1 and 24 h of HFD. Since there was no detectable dose-dependency regarding treatment duration, we assume that the naturally intermittent diurnal feeding behavior sustains BBB leakage upon HFD exposure of more than 1 h. Whether BBB permeability further increases with HFD treatment longer than 24 h remains to be elucidated with the *in vivo* imaging approach. Our results also suggest that BBB leakage following acute HFD exposure is region-specific. The somatosensory cortex, where we detected FITC-dextran leakage using 2-photon microscopy, represents a functionally discrete and spatially restricted area. In contrast, when homogenizing the entire cortex to analyze NaF and doxorubicin by spectrophotometry, the leakage was not detectable after 1 h of HFD. This method likely dilutes any localized leakage effects and has a lower spatial resolution to detect permeability changes in specific cortical subregions compared to 2-photon microscopy. However, both the analysis of NaF and doxorubicin showed leakage specifically in the hippocampus; this region has a more consistent structural organization compared to the cortex, which may contribute to more robust BBB leakage that is detectable even with a less spatially resolved method. Additionally, existing literature suggests that the hippocampal BBB is inherently more permeable to peripheral blood components compared to other brain regions [23]. Furthermore, its proximity to the dorsal third and lateral ventricles may result in greater exposure of the hippocampus to circulating factors, which could further contribute to increased permeability in this region [24]. Compared to other brain regions, the hypothalamus showed higher tracer infiltration rates of both NaF and doxorubicin. Since the hypothalamus is an area with a fenestrated BBB [15,21], the higher NaF and doxorubicin permeation observed there compared to other regions confirms the reliability of our method for estimating BBB permeability. Additionally, we likely encounter a ceiling effect in this region; because baseline leakage is already higher than in other areas, HFD treatment does not further increase it.

It is important to note that volatile agents such as iso- and sevoflurane also disrupt the BBB [25]. For this reason, we have used an injectable anesthetic cocktail consisting of medetomidine, midazolam, and fentanyl in our study. Administration of dexmedetomidine, an active metabolite of medetomidine, has been shown to prevent BBB disruption [26] and repeated injection of midazolam also has protective effects against BBB leakage [27]. Thus, our results are even likely to underestimate the effects of acute HFD.

Putative mechanisms of rapid HFD-induced BBB leakage could be hypothesized based on experiments with short-term treatment periods: an increase in inflammatory markers in the murine hippocampus and hypothalamus were reported after 48 and 24 h of HFD, respectively [18,28]. However, many regulators of BBB permeability have been identified in addition to inflammatory cytokines, including nitrous oxide, calcium influx, or hemodynamic changes [2]. Overall, the dynamic modulation of the BBB could also serve a physiological purpose, for example allowing faster transport of peptide hormones that regulate feeding and nutrient metabolism into the brain [21,29]. With this in mind, acute HFD could also influence BBB permeability through novel and specific mechanisms. It is possible that the components of HFD, especially the different nutrients available after its digestion could be responsible for the BBB leakage after acute HFD; either fats, amino acids, micronutrients or peptides released. Another intriguing hypothesis is that a rise in osmolality after feeding induces leakage in the BBB, as has been shown for mannitol infusions in the past [30]. Thus, several possible mediators of the rapid HFD-induced BBB alteration have to be explored next.

Finally, the striking finding that acute HFD-induced BBB leakiness for two exogenous molecules of different sizes led to the clinically relevant question of whether acute dietary choices could also impact the brain delivery of drugs that do not penetrate the BBB well. We demonstrated a 2-fold increased concentration of the chemotherapeutic agent doxorubicin in the hippocampus after eating HFD for 1 h. This could be of relevance in the context of drug treatments. For instance, if the target is a brain tumor, eating high amounts of energy-dense food could potentially enhance drug delivery. On the other hand, increased concentrations of a neurotoxic drug in healthy brain tissue might lead to severe side effects. Here, we provide the foundation for a new line of research not only exploring how acute dietary stimuli might modulate BBB permeability and influence pharmacotherapy outcomes but also for identifying the mechanism behind this phenomenon and potentially developing innovative therapeutic approaches for brain pathologies.

## 5 Limitations

This study was conducted in mice, which limits the direct translation of our findings to humans, particularly in the context of brain tumors and clinical treatments. We focused on unveiling and validating the effects of acute HFD exposure on BBB permeability but did not investigate the underlying mechanisms, which need to be addressed in future studies. All experiments were performed in anesthetized animals using the same protocol throughout, as discussed earlier. The potential influence of anesthesia, as well as factors such as circadian stage and wakefulness, should be considered when interpreting the results and planning future studies.

## CRediT authorship contribution statement

Conceptualization: EL, AZ, VS, and AKG; Methodology: EL and AKG; Investigation: EL and AKG; Writing—original draft: EL and AKG; Writing—review and editing: EL, AZ, VS, and AKG; Funding acquisition: EL and AKG; Supervision: EL and AKG. All authors contributed to the final version of the manuscript.

## Declaration of Generative AI and AI-assisted technologies in the writing process

No AI or AI-assisted technologies were used.

## Data availability

All requests should be sent to the corresponding authors AKG or EL. The datasets generated during and/or analyzed during the current study are available from the corresponding authors on reasonable request.

## Declaration of competing interest

The authors declare the following financial interests/personal relationships which may be considered as potential competing interests:

Anne-Kathrin Gellner reports a relationship with Novartis Pharma GmbH that includes: speaking and lecture fees and travel reimbursement. If there are other authors, they declare that they have no known competing financial interests or personal relationships that could have appeared to influence the work reported in this paper.
