# Supplementary material for: Acute high-fat high-sugar diet rapidly increases blood-brain barrier permeability in mice
Source: J Nutr Health Aging. 2025 May 16;29(7):100574. doi: 10.1016/j.jnha.2025.100574 (PMC12172964; doi:10.1016/j.jnha.2025.100574)
Supplement: Supplementary file 1 [file mmc1.docx]

## Supplementary figures

**Supplementary table 1. Mouse cohorts and their ages used in the study**

| **Cohort** | **Tracer, size** | **Method to quantify the BBB permeability** | **Experimental treatments** | **Age of mice** |
| --- | --- | --- | --- | --- |
| 1 | Sodium fluorescein (NaF), 376 Da | Spectrophotometrical measurements in homogenized brain regions | Standard chow control or 1 h HFD (between-subject design) | 10 months |
| 2 | FITC-dextran, 70 000 Da | *In vivo* 2-photon imaging with cranial window above the somatosensory cortex | Standard chow conrol, 1 h HFD, 24h HFD (within-subject design, pseudorandomized treatments) | 4-6 months |
| 3 | Doxorubicin, 580 Da | Spectrophotometrical measurements in homogenized brain regions | Standard chow control or 1 h HFD (between-subject design) | 4-5 months |

**
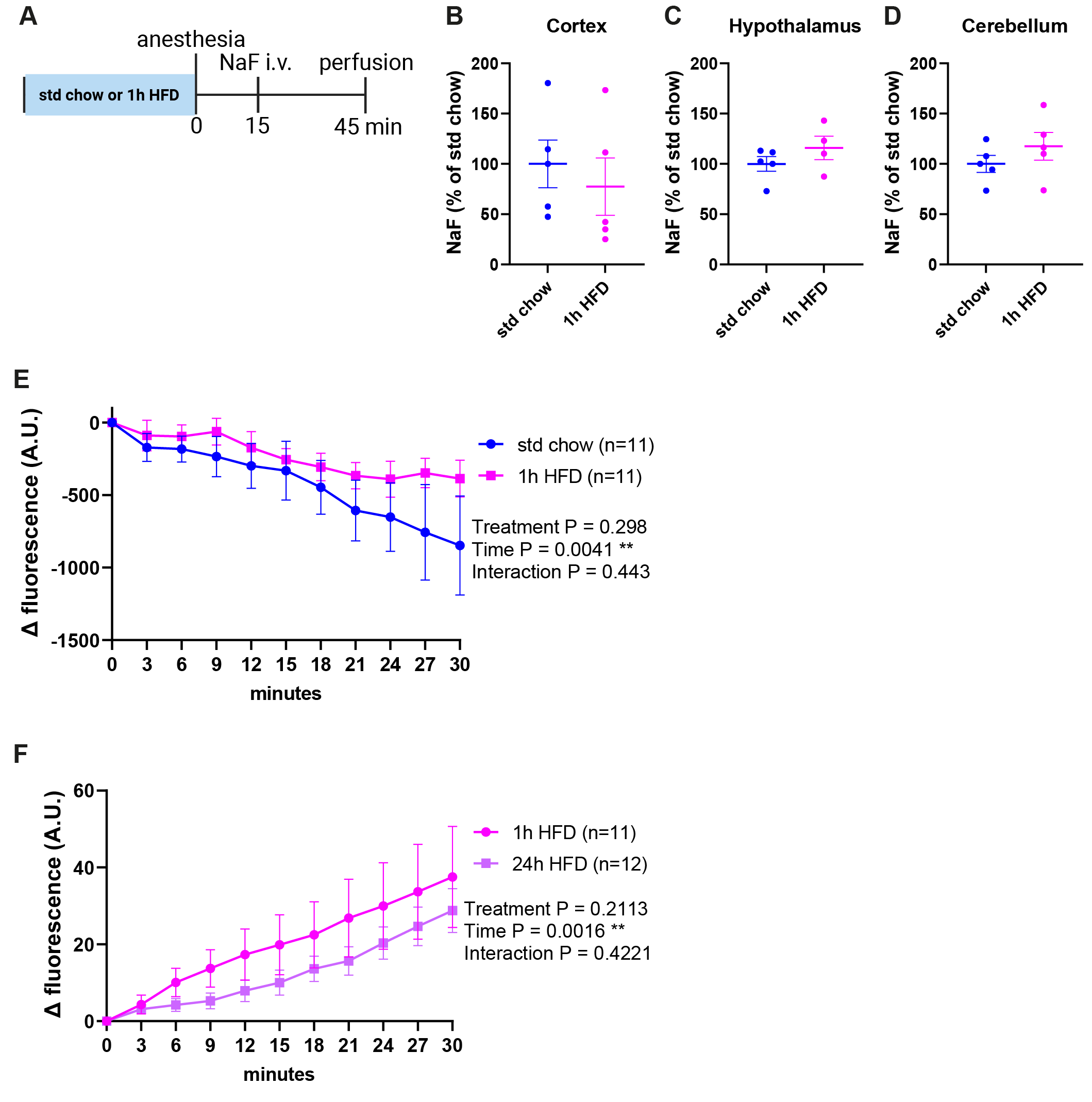
**

**Supplementary Figure 1. Unaltered leakage of small molecular weight tracer sodium fluorescein (NaF) (376 Da) from blood into the brain tissue in cortex, hypothalamus, and cerebellum after 1 hour of high-fat high-sugar diet (HFD) exposure.**

**(A)** Scheme showing the design of the experiment. Relative NaF concentrations were not altered (t(8) = 0.610, p = 0.559) after 1h of HFD in cortex **(B)**, hypothalamus (t(7) = 1.22, p = 0.263) **(C),** or cerebellum (t(8) = 1.09, p = 0.308) **(D)**. Changes of intravascular FITC-dextran levels in the somatosensory cortex are significant over the time course of imaging but do not differ by treatment; treatment F(1, 11) = 1.95, p = 0.298, time F(2.1, 23.1) = 6.866, p = 0.0041, time x treatment interaction F(1.922, 16.91) = 0.844, p = 0.443 **(E)**. The leakage of FITC-dextran into the somatosensory cortex is not statistically different when comparing 1- and 24-hour exposure to HFD; treatment F(1, 11) = 1.762, p = 0.211, time F(1.167, 12.83) = 14.6, p = 0.0016, time x treatment interaction F(1.508, 14.93) = 0.835, p = 0.422 **(F)**. Error bars represent mean ± SEM.


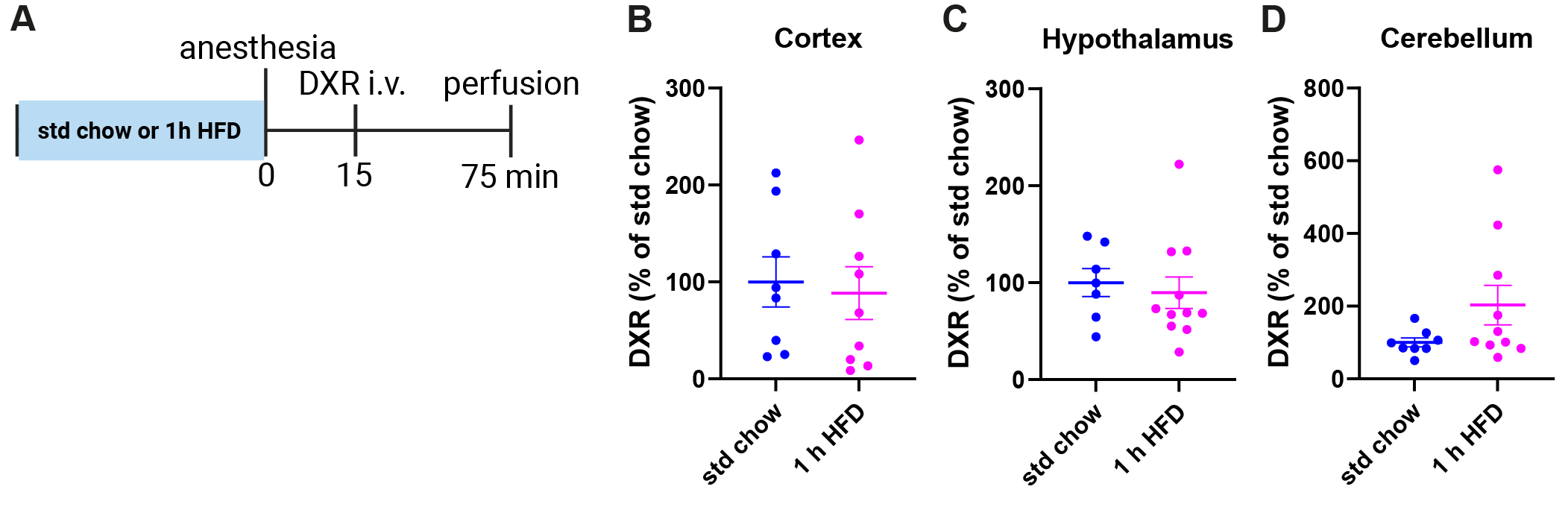


**Supplementary Figure 2. Unaltered leakage of doxorubicin (580 Da) from blood into the brain tissue of cortex, hypothalamus, and cerebellum after 1 hour of high-fat high-sugar diet (HFD) exposure.**

**(A)** Scheme showing the design of the experiment. Relative DXR concentrations were not altered after 1 hour of HFD in cortex (t(15) = 0.309, p = 0.762) **(B)**, hypothalamus (U = 29, p = 0.425) **(C),** or cerebellum (U = 23, p = 0.146) **(D)**. Error bars represent mean ± SEM.
